# Genome-based analysis for the bioactive potential of *Streptomyces yeochonensis* CN732, an acidophilic filamentous soil actinobacterium

**DOI:** 10.1186/s12864-020-6468-5

**Published:** 2020-02-03

**Authors:** Adeel Malik, Yu Ri Kim, In Hee Jang, Sunghoon Hwang, Dong-Chan Oh, Seung Bum Kim

**Affiliations:** 1 Department of Microbiology and Molecular Biology, Chungnam National University, Daejeon, 34134 Republic of Korea; 2 Natural Products Research Institute, College of Pharmacy, Seoul National University, Seoul, 08826 Republic of Korea

**Keywords:** *Streptomyces yeochonensis*, Neutrotolerant acidophilic, Secondary metabolite, Core genome, Singletons, CAZyme, Sortase

## Abstract

**Background:**

Acidophilic members of the genus *Streptomyces* can be a good source for novel secondary metabolites and degradative enzymes of biopolymers. In this study, a genome-based approach on *Streptomyces yeochonensis* CN732, a representative neutrotolerant acidophilic streptomycete, was employed to examine the biosynthetic as well as enzymatic potential, and also presence of any genetic tools for adaptation in acidic environment.

**Results:**

A high quality draft genome (7.8 Mb) of *S. yeochonensis* CN732 was obtained with a G + C content of 73.53% and 6549 protein coding genes. The in silico analysis predicted presence of multiple biosynthetic gene clusters (BGCs), which showed similarity with those for antimicrobial, anticancer or antiparasitic compounds. However, the low levels of similarity with known BGCs for most cases suggested novelty of the metabolites from those predicted gene clusters. The production of various novel metabolites was also confirmed from the combined high performance liquid chromatography-mass spectrometry analysis. Through comparative genome analysis with related *Streptomyces* species, genes specific to strain CN732 and also those specific to neutrotolerant acidophilic species could be identified, which showed that genes for metabolism in diverse environment were enriched among acidophilic species. In addition, the presence of strain specific genes for carbohydrate active enzymes (CAZyme) along with many other singletons indicated uniqueness of the genetic makeup of strain CN732. The presence of cysteine transpeptidases (sortases) among the BGCs was also observed from this study, which implies their putative roles in the biosynthesis of secondary metabolites.

**Conclusions:**

This study highlights the bioactive potential of strain CN732, an acidophilic streptomycete with regard to secondary metabolite production and biodegradation potential using genomics based approach. The comparative genome analysis revealed genes specific to CN732 and also those among acidophilic species, which could give some insights into the adaptation of microbial life in acidic environment.

## Background

Within the phylum Actinobacteria, the genus *Streptomyces* represents one of the most diverse groups primarily found in soil and aquatic habitats and playing a substantial role in carbon recycling [1]. Streptomycetes are filamentous, sporulating Gram-positive bacteria capable of metabolizing a broad range of carbon sources as well as biosynthesizing several secondary metabolites with industrial implications [2]. Majority of the compounds of microbial origin discovered till date with antibiotic, antitumor, or immunosuppressive activities have been derived from *Streptomyces* [3]. Such bioactive compounds are produced by biosynthetic gene clusters (BGCs) that consist of genes arranged in close proximity within the bacterial genomes [4]. Based on their products, BGCs are in general classified as non-ribosomal peptide synthetases (NRPSs), polyketide synthases (PKSs), and those for saccharides, terpenoids, lanthipeptides and many others. The diversity of these BGCs could be further enhanced by the combination of two or more such clusters to form hybrid BGCs. NRPS, PKS and their hybrids have attracted more attention because of the diversity of unique structures that are produced from these BGCs as a result of highly regulated, step-wise activity of enzymes localized in such clusters [5]. It was suggested that *Streptomyces* might produce as many as 100,000 antimicrobial metabolites, out of which only a little percentage has been identified [6]. Recognizing the concern that application of currently used antibiotics might become inefficacious against numerous pathogens because of the increase in number of antimicrobial resistant microbes, search for novel strains of *Streptomyces* is thus crucial to help fill the critical need for new antibiotics [7].

In addition to their ability for secondary metabolite production, streptomycetes are also considered as key players in the decomposition of plant biomass [8]. The bulk of the energy in this plant biomass is stored in plant cell walls, mainly in the form of polysaccharides such as cellulose and hemicellulose. Similarly, chitin is the second most abundant polysaccharide in nature, next only to cellulose, and is found in the exoskeleton of insects, fungi, yeast, and algae, as well as in the internal structures of other vertebrates [9]. The formation and breakdown of such substances is controlled by various enzymes known as carbohydrate-active enzymes (CAZymes) [10]. From industrial perspective, breakdown of such biomass is very challenging because of the limitations of having efficient enzymes that could economically hydrolyze these complex carbohydrates [11]. Microorganisms with biomass-degrading capabilities offer a great promise to breakdown complex glycans into simple sugars [1]. However, only a limited number of bacteria and fungi have developed the ability to efficiently breakdown these insoluble polymers [12]. It has been proposed that species of *Streptomyces* are capable of efficiently degrading these complex sugars, and hence could be used for biotechnological applications [1, 13, 14].

Acidophilic species are among the species considered to have high antimicrobial potential [15], and yet only a limited attention has been given to their secondary metabolite biosynthesis that still remains mostly unexplored [16]. In fact only a minor proportion of the species among *Streptomyces*, as only 6 species out of over 700 species are known as acidophilic [bacterio.net/streptomyces.html], and no studies on their bioactive potential have been conducted to date. Actinobacteria from acidic soils are believed to be better sources of polyketides such as polyether ionophores that show broad activities and striking effectiveness against drug-resistant bacteria and parasites [17].

In this work, we report a genome based study on the bioactive potential of a representative neutrotolerant acidophilic streptomycete, *Streptomyces yeochonensis* CN732 [18]. The strain is a Gram-positive, non-motile and aerobic actinobacterium from soil that forms largely branched substrate and aerial mycelia. With a focus to identify genomic features related to the secondary metabolite production, efforts were made to explore the enrichment of enzymes specific to this streptomycete as compared to some well-known *Streptomyces* strains for which genome data are available. The comparative genomic analysis reveals that strain CN732 has a collection of genes encoding enzymes necessary for secondary metabolites and biomass degradation, and also that there are a range of genes specific for neutrotolerant acidophilic species. The roles of such enzymes in the biosynthetic clusters were also examined.

## Results and discussion

### General genomic features and phylogeny of *Streptomyces yeochonensis* CN732

**Fig. 1:**
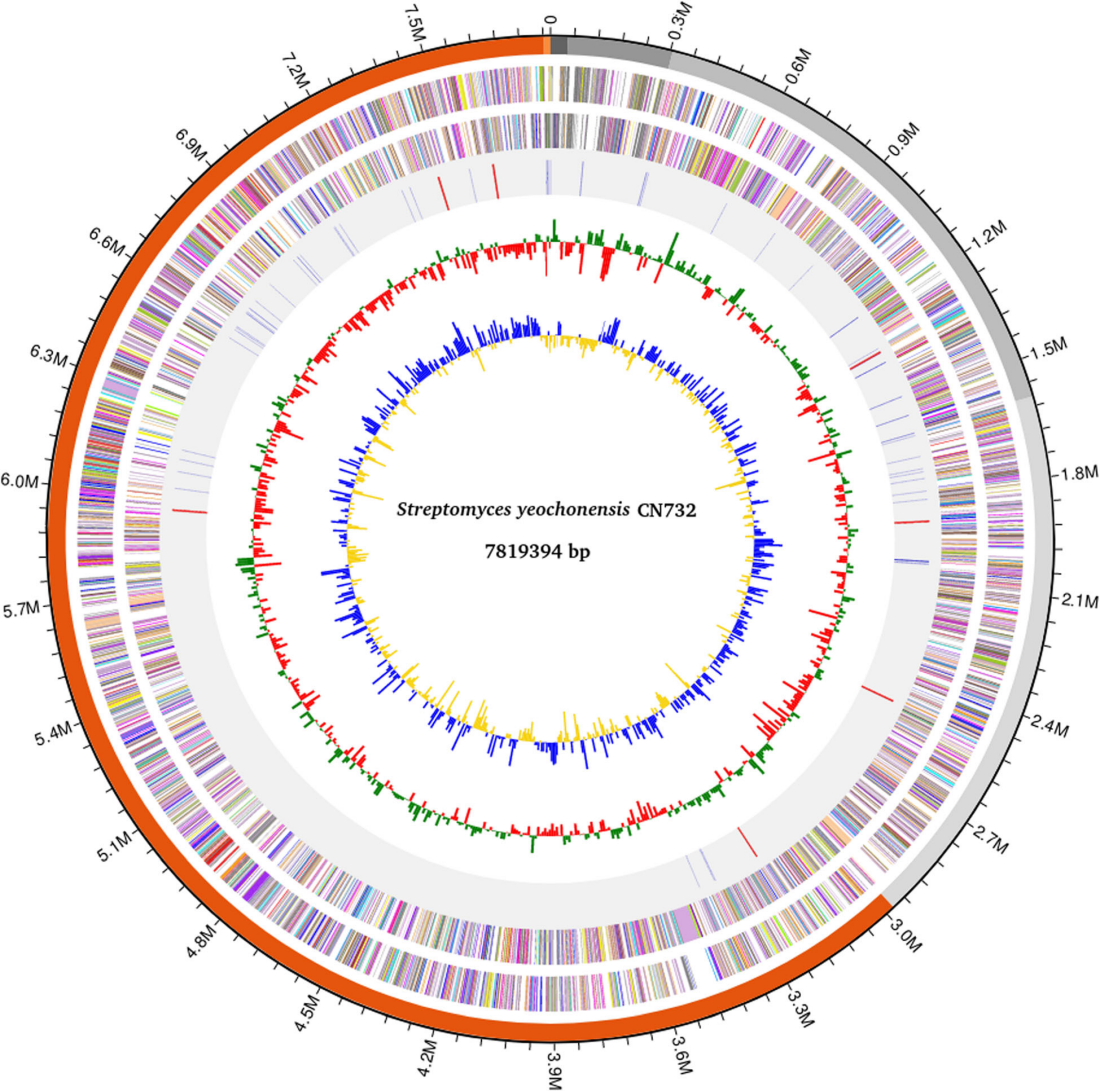
Circular map of the *S. yeochonensis* CN732 genome retrieved from EZBioCloud [https://www.ezbiocloud.net/]. Description of each circle is represented from the outermost circle to the innermost. (1) All the 6 contigs are shown as separate colors. (2 and 3) Tick marks representing the predicted CDS on the positive strand and negative strands. Each CDS is color-coded by its COG category (http://help.bioiplug.com/cog-colors/). (4) Positions of rRNAs and tRNAs are highlighted. (5) GC Skew. (6) GC Ratio

A high quality draft genome sequence consisting of 6 contigs was obtained for strain CN732 (Fig. 1). The total stretch of these contigs was 7,819,394 bp, and the contig length of N50 was 4,825,649 bp. An average G + C content of 73.56% was observed in strain CN732, which is also the highest among all the strains used in this study. A total of 6549 protein coding genes (CDS), 109 pseudogenes, 65 tRNA and 21 rRNA genes were predicted by RAST annotation. Table 1 provides the overview of the genomic features of strain CN732 and its comparison with other selected *Streptomyces* species for which genome information is available. Overall, the average G + C content of acidophilic strains, namely *S. yeochonensis* CN732, *S. guanduensis* CGMCC 4.2022, *S. yanglinensis* CGMCC 4.2023, *S. rubidus* CGMCC 4.2026 and *S. paucisporeus* CGMCC 4.2025 was slightly higher (72.89% ±0.52) as compared to the non-acidophilic *Streptomyces* (71.68 ± 0.83). Moreover, very few number of rRNAs were observed in the genomes of almost all acidophilic *Streptomyces* except in the case of strain CN732.

**Table 1:** General genomic features of *Streptomyces yeochonensis* CN732 and other species used in this study

Strain	BioProject Accession	Size (Mbp)	No. of Contigs	% G+C	CDS	tRNA	rRNA
*S. yeochonensis* CN732	PRJNA234789	7.82	6	73.6	6,549	65	21
*S. albus* DSM 41398	PRJNA271625	8.38	1	72.6	6,923	65	18
*S. avermitilis* MA-4680	PRJNA189	9.12	2	70.7	8,106	68	12
*S. bingchenggensis* BCW-1	PRJNA46847	11.94	1	70.8	10,313	64	18
*S. coelicolor* A3(2)	PRJNA242	9.05	3	72.0	8,325	65	12
*S. collinus* Tu 365	PRJNA171216	8.38	3	72.6	7,336	71	18
*S. davawensis* JCM 4913	PRJEB184	9.56	2	70.6	8,696	69	18
*S. fulvissimus* DSM 40593	PRJNA192408	7.91	1	71.5	7,081	72	18
*S. glaucescens* GLA.O	PRJNA260814	7.62	2	72.9	6,719	66	18
*S. griseus* NBRC 13350	PRJNA20085	8.55	1	72.2	7,294	66	12
*S. nodosus* ATCC 14899	PRJNA259817	7.71	1	70.8	6,875	66	18
*S. rapamycinicus* NRRL 5491	PRJNA207502	12.70	1	70.6	10,393	64	1
*Streptomyces* sp. SirexAA-E	PRJNA38225	7.41	1	71.8	6,808	63	18
*S. venezuelae* ATCC 10712	PRJNA62209	8.23	1	72.4	7,409	65	20
*S. vietnamensis* GIM4.0001	PRJNA244969	9.15	2	72.0	8,292	71	21
*S. guanduensis* CGMCC 4.2022	PRJEB16229	8.22	33	73.1	7,183	61	6
*S. paucisporeus* CGMCC 4.2025	PRJEB18367	8.16	79	72.22	7,380	62	8
*S. rubidus* CGMCC 4.2026	PRJEB16923	9.00	112	72.91	8,120	66	7
*S. yanglinensis* CGMCC 4.2023	PRJEB16703	9.59	44	72.6	8,530	64	7

**Fig. 2:**
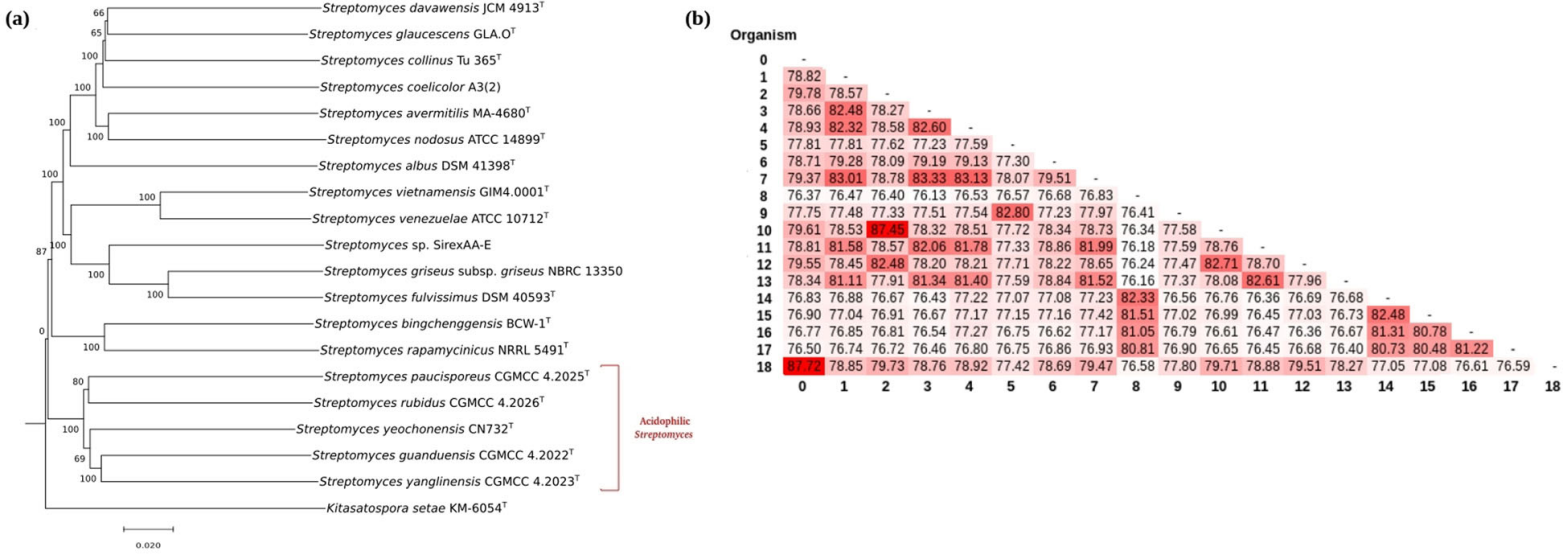
Relationship of *S. yeochonensis* CN732 with 14 neutrotolerant and 4 acidophilic *Streptomyces* based on, **a** Whole genome-based tree inferred with FastME from GBDP distances calculated from the genome sequences. The branch lengths are scaled in terms of GBDP distance formula *d5*. Numbers above branches are GBDP pseudo-bootstrap support values from 100 replications. The tree was rooted at the midpoint and *K. setae* KM-6054^T^ was used as an out-group. **b** Average nucleotide identity (ANI) scores between all *Streptomyces* (0 = *S. venezuelae* ATCC 10712, 1 =*S. coelicolor* A3(2), 2 = *S. griseus* subsp. *griseus* NBRC 13350, 3 = *S. davaonensis* JCM 4913, 4 = *S. collinus* Tu 365, 5 = *S. rapamycinicus* NRRL 5491, 6 = *S. albus* DSM 41398, 7 = *S. glaucescens* GLA.O, 8 = *S. yanglinensis* CGMCC 4.2023, 9 = *S. bingchenggensis* BCW-1, 10 = *S. fulvissimus* DSM 40593, 11 = *S. avermitilis* MA-4680, 12 = *Streptomyces* sp. SirexAA-E, 13 = *S. nodosus *ATCC 14899, 14 = *S. guanduensis* CGMCC 4.2022, 15 = *S. yeochonensis* CN732, 16 = *S. rubidus* CGMCC 4.2026, 17 = *S. paucisporeus* CGMCC 4.2025, 18 = *S. vietnamensis* GIM4.0001) strains

The taxonomic position of strain CN732 (Additional file 1: Figure S1) was previously established within the genus *Streptomyces* [18]. This was further verified by a genome-based phylogeny of strain CN732 and other well known *Streptomyces* species, in which strain CN732 was clustered with the four acidophilic *Streptomyces* species (Fig. 2a). This was also supported by the average nucleotide identity (ANI) scores, as the ANI values between *S. yeochonensis* CN732 and other acidophilic *Streptomyces* species ranged between 80.48~82.48%, but the values with other *Streptomyces* species ranged between 76.45 and 77.42% (Fig. 2b).

### Biosynthetic gene clusters for secondary metabolites of strain CN732

A total of 22 secondary metabolite producing gene clusters were identified, including 2 NRPS (non-ribosomal peptide synthetase) type, 3 PKS (polyketide synthase) type and 3 hybrid clusters, namely 2 Type 1 PKS-NRPS and 1 Type 1 PKS-butyrolactone type biosynthetic clusters (Table 2). Terpene biosynthesis related clusters were the most abundant type of clusters observed in the CN732 genome. Out of the 22 potential biosynthetic clusters, 15 exhibited some level of similarities with known BGC whereas 7 clusters represented orphan BGCs for which no known homologous gene clusters [19] could be identified. Notably, non-ribosomal peptide synthetase and melanin type clusters shared similarity with those for antibacterial compounds, whereas the majority of polyketide, peptide or hybrid type clusters shared similarity with those for anticancer or antiparasitic compounds. However, the levels of similarity were fairly low in most cases, which suggests the novelty of the possible metabolites from those predicted gene clusters.

**Table 2:** List of putative secondary metabolite producing biosynthetic clusters as predicted by antiSMASH

Cluster	Size (bp)	Most similar known biosynthetic cluster^a^	MIBiG BGC-ID
*Terpenes*:			
1	22,253	-	-
10	21,077	2-Methylisoborneol (100%)	BGC0000658_c1
14	26,642	Hopene (69%)	BGC0000663_c1
20	21,158	-	-
*NRPS*:			
2	64,175	Laspartomycin (9%)	BGC0000379_c1
18	56,142	Enduracidin (6%)	BGC0000341_c1
*Siderophores*:			
4	11,797	Desferrioxamine B (80%)	BGC0000941_c1
9	16,454	-	-
*PKS*:			
19 (type 1)	45,946	Maduropeptin (22%)	BGC0001008_c1
5 (type 2)	42,525	Spore pigment (83%)	BGC0000271_c1
13 (type 3)	41,056	Alkylresorcinol (100%)	BGC0000282_c1
*Peptides*:			
8 (lantipeptide)	24,416	-	-
11 (thiopeptide)	26,162	Actinomycin (10%)	BGC0000296_c1
16 (thiopeptide)	33,062	Grincamycin (5%)	BGC0000229_c1
*Butyrolactones*:			
12	10,840	Himastatin (8%)	BGC0001117_c1
17	10,938	-	-
*Hybrids*:			
7 (T1PKS-NRPS)	92,837	Meilingmycin (13%)	BGC0000093_c1
22 (T1PKS-NRPS)	64,965	Bleomycin (6%)	BGC0000963_c1
15 (T1PKS-butyrolactone)	53,890	Tirandamycin (13%)	BGC0001052_c1
*Others*:			
3 (nucleoside)	20,387	-	-
6 (melanin)	10,408	Istamycin (5%)	BGC0000700_c1
21 (bacteriocin)	10,807	-	-

^a^The percentage in parentheses indicate the number of genes showing similarity to the corresponding known biosynthetic cluster

**Fig. 3:**
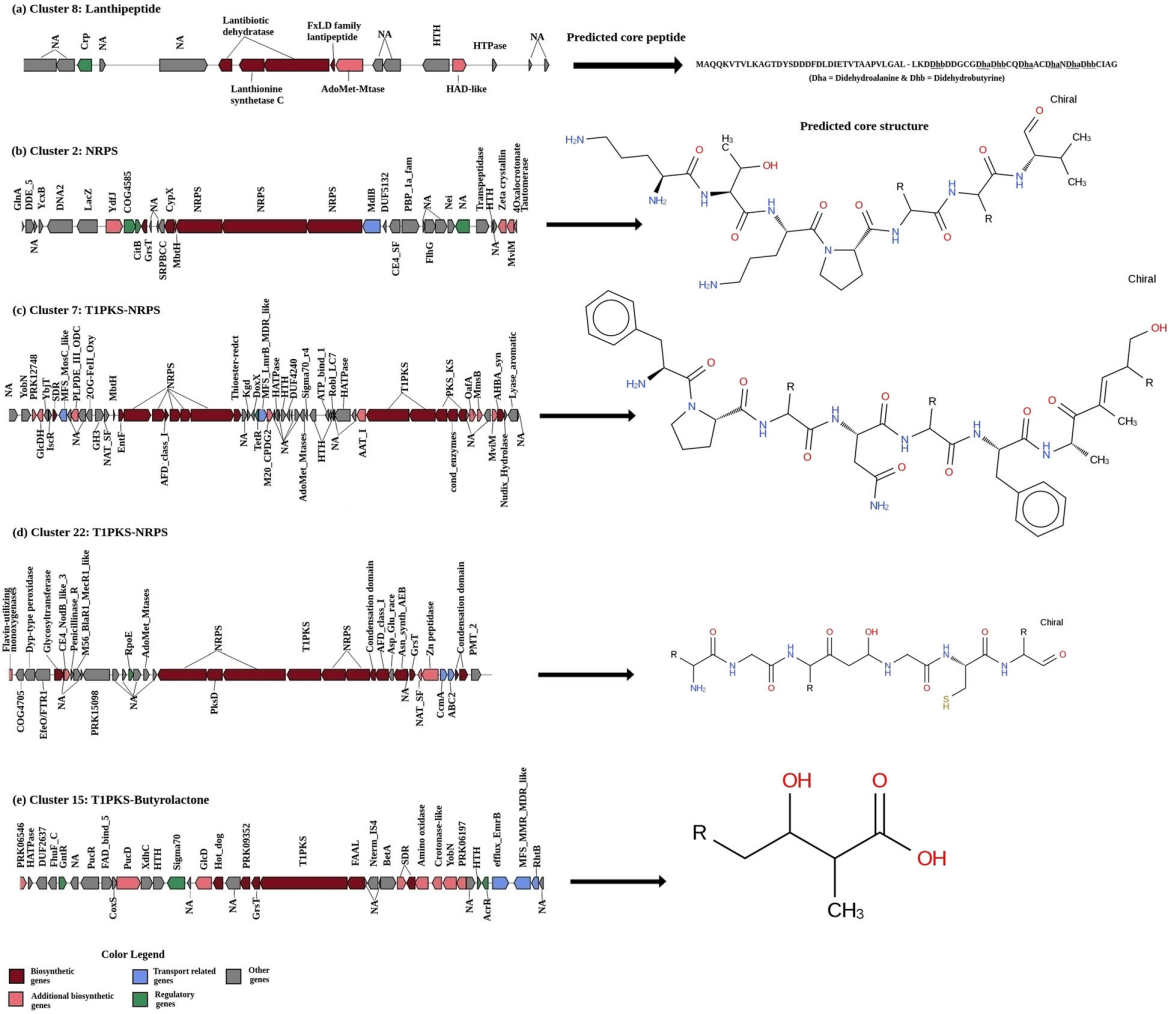
antiSMASH predicted biosynthetic gene clusters and their predicted core structures for **a** lanthipeptide, **b** NRPS, **c**, **d** Type 1 PKS-NRPS, and **e** Type 1 PKS-Butyrolactone clusters from *S. yeochonensis* CN732 genome

There were at least 4 clusters for which a core structure was predicted. These include 2 Type 1 PKS-NRPS, 1 NRPS, and 1 Type 1 PKS-butyrolactone gene clusters. Furthermore, a core peptide representing a putative class I lanthipeptide was also predicted (Fig. 3a). This lanthipeptide cluster is the only orphan biosynthetic gene cluster in strain CN732 for which a structure was predicted by antiSMASH. The class I lanthipeptides are synthesized by the enzymatic action of a dehydratase (LanB) and a cyclase (LanC) [20], both of which are present in cluster 8. Moreover, the zinc-binding motif (Cys-Cys-His/Cys) present in LanC enzymes [21] was also well conserved in the putative LanC enzyme from CN732.

In addition to the presence of core biosynthetic genes, there were at least 13 clusters (clusters 1, 2, 5, 7, 10, 13, 15–19, 21, 22) in CN732 genome that contained genes for transcription regulation and transport. Similarly, about 23 genes encoding various CAZymes were identified in 16 biosynthetic clusters (clusters 1, 3–4, 6–9, 12, 14, 16–17, and 20–24). These CAZymes consisted of one or more CAZy [10] family domains and include glycosyl hydrolases (GHs), glycosyltransferases (GTs), carbohydrate esterases (CEs), and few redox enzymes having auxiliary activities (AAs) that work simultaneously with CAZymes. Genes containing carbohydrate binding modules (CBMs) were also observed in some clusters (Additional file 2: Table S1). Previous studies have highlighted the role of these CAZymes in the biosynthesis of antibiotics such as oleandomycin [22] and spiramycin [23]. Several biosynthetic molecules of microbial origin attribute their biological activities to the attached glycan moieties [24], which if altered could have a serious impact on the selectivity, activity and pharmacokinetic properties [25, 26] of the parent compound. Therefore, in addition to the presence of core PKS and NRPS genes, the secondary metabolite producing clusters detected in CN732 genome also consisted of diverse CAZymes required for imparting biological activities.

### Biosynthetic gene clusters with predicted core structures of strain CN732

#### NRPS gene cluster

The NRPS cluster 2 with a predicted core structure observed in strain CN732 consisted of 25 domains which included 6 condensation (C) domains, and 7 domains each of adenylation (A) and peptidyl carrier protein (PCP, also known as a thiolation (T) domain) domains. All these three types of domains are the essential components of an NRPS system and catalyze primary steps in the formation of a peptide product [27]. Among these, incorporation of substrates at the A domain in each module imparts diversity to NRPS products [4]. The remaining 5 depicted N-methylation (NMT), thioesterase (TE) and enoylreductase (ER) domains, respectively. The predicted peptide from this cluster represented a backbone structure of (Orn-Thr) + (Orn-Pro-NRP-Bht|Tyr) + (Val), where Orn denotes ornithine and bht = β-hydroxy-tyrosine (Fig. 3b). Based on the antiSMASH analysis, only a limited number of genes present in this cluster exhibited similarity (9%) to the known homologous gene cluster of laspartomycin biosynthesis [28]. Laspartomycins are 11 amino acid peptide antibiotics synthesized by *lpm* BGC from *Streptomyces viridochromogenes.* The *lpm* cluster consists of 21 open reading frames (ORFs) which include four NRPS genes, four regulatory genes, four lipid tail biosynthesis and attachment genes, and three putative self-resistance or exporter genes. In contrast, cluster 2 from strain CN732 consisted of only three NRPS genes all of which differed from the *lpm* cluster of *S*. *viridochromogenes* in their domain structure and organization. For example, in addition to the differences in the number of C-A-T domains, the epimerization (E) domains were absent in two of these NRPS enzymes that were present in two out of four NRPS enzymes from *S*. *viridochromogenes*. However, the regulatory genes that code for signal transduction histidine kinases as well as other transcriptional regulators were present. Therefore, it is expected that the putative biosynthetic compound from this NRPS gene cluster 2 may represent a novel chemical structure.

#### PKS-NRPS hybrid gene clusters

The genome of CN732 contained two potential Type 1 PKS-NRPS hybrid clusters (clusters 7 and 22), which are probably the largest among all 22 predicted clusters with the sizes of approximately 93 kbp and 65 kbp, respectively. In general, each Type 1 PKS module consists of at least one domain each of a ketosynthase (KS), acyltransferase (AT), and acyl carrier protein (ACP), although additional domains such as dehydratase (DH), enoylreductase and ketoreductase (KR) may also be present [29]. The modular structure and domain organization of the core biosynthetic genes of both the hybrid clusters were observed to be different from each other. Similarly, the predicted core peptide structures from these hybrid clusters were also different (Fig. 3c and d). Specifically, a hybrid cluster (cluster 7) consisted of two additional TD (thioester reductase domain of alpha aminoadipate reductase Lys2 and NRPSs), 2 aspartate aminotransferase (aminotran) and one epimerase (E) domains. In addition to the differences observed at the domain level of core biosynthetic genes, differences in the number and type of additional biosynthetic genes, transport and regulatory genes were also observed. Moreover, the number of genes that exhibited homology to known gene clusters for clusters 7 and 22 were 13 and 6% with BGCs for meilingmycin and bleomycin, which are known for antiparasitic and anticancer activities respectively. The known meilingmycin BGC essentially consists of multiple PKS genes [30] as compared to the hybrid Type 1 PKS-NRPS cluster 2 of strain CN732 which in turn consisted of at least two NRPS genes in addition to two PKS genes. In contrast, the known bleomycin BGC from *Streptomyces verticillus* [31] consisted of multiple NRPS genes and a single PKS. Although cluster 22 of strain CN732 also consisted of multiple NRPS genes, the number was lesser than the known bleomycin BGC. Moreover, a significantly different domain architecture of these NRPS genes was observed in cluster 22. One of the NRPS enzymes in cluster 22 contained an additional KR and DH domains besides C, A and T domains. The architecture of single PKS genes also differed in both clusters. For example, the PKS from bleomycin cluster consisted of KS, AT, cMT, KR and PCP domains (in that order) whereas the domains present in a single PKS gene of cluster 22 contained KS, AT, DHt, KR and PCP domains.

#### Other biosynthetic gene clusters

In addition to the two hybrid Type 1 PKS-NRPS clusters discussed above, one Type 1 PKS-butyrolactone hybrid cluster (cluster 15) of about 54 kbp was also detected (Fig. 3e). This cluster also exhibited limited similarity (13%) with a hybrid Type 1 PKS-NRPS BGC from *Streptomyces* sp. 307–9 which is known to produce tirandamycin, a group of compounds showing antiparasitic, antifungal or antibacterial activities [32]. Tirandamycin BGC consists of three PKS and one NRPS proteins, in addition to proteins involved in tailoring, self-resistance and regulatory steps, whereas cluster 15 consisted of only one PKS protein and lacked any NRPS coding gene. However, several additional biosynthetic genes such as dehydrogenases and oxidases, transport-related and regulatory genes were also observed in this cluster. These results again imply the potential diversity of hybrid compounds produced from this strain. Because of their extended biosynthetic capabilities, a diverse array of biosynthetic compounds can be produced from such clusters, and therefore, these hybrid systems have gained much attention from scientific community [33–35]. All of the above discussed clusters also contained at least two or more CAZy domains.

Furthermore, the annotation of CN732 genome also led to the identification of at least 7 additional genes related to polyketide biosynthesis known as polyketide cyclases (PCs) or SnoaL-like polyketide cyclases. Among these PCs, only two were detected in cluster 2 (Type 2 PKS), whereas one PC was identified to be a singleton. PCs have been well characterized within the genus *Streptomyces* and are known to catalyze the last ring closure step in the course of biosynthesis of a variety of compounds such as anthracyclines [4], which include some of the most powerful groups of aromatic polyketide antibiotics, e.g., doxorubicin and daunorubicin [36, 37]. Therefore, taken together, the limited similarities shared between the antiSMASH clusters detected in the CN732 genome and their corresponding biosynthetic genes as compared to the known secondary metabolite producing gene clusters indicate putative uniqueness of the compounds that may be produced by this strain.

### Antimicrobial potential of strain CN732

The PCR based detection yielded positive results for both NRPS and PKS 1 genes (Additional file 3: Figure S2), although other bands were also seen in both cases. This may be the result of non-specific amplifications due to the multiple degenerate positions for the forward and reverse primers in both primer sets (3 and 2 positions for NRPS, and 7 and 6 positions for PKS 1 respectively). Strain CN732 exhibited antagonistic activity against a range of microbes including Gram positives, Gram negatives and yeasts, which proves a high antimicrobial potential of this strain (Additional file 2: Table S2). The activity was dependent on the media employed for tests, as highest proportions of positive results were observed for SNA medium and PEC medium whereas no positive results could be observed for Bennett’s medium and Mueller-Hinton medium. Such differences might be attributed to different degrees of growth on these media.

### Secondary metabolites from strain CN732

The combined high performance liquid chromatography-mass spectrometry (HPLC-MS) analysis confirmed production of various compounds. One of the compounds yielded a UV spectrum with absorption maxima at 218, 238, 353 and 386 nm, and a positive mass spectrum with an m/z ratio of 243.1, which was identified as lumichrome with a molecular mass of 242.1 (Additional file 4: Figure S3). Lumichrome (7,8-dimethylalloxazine) is a photodegradation product of riboflavin, and known as an effective photosensitizer and fluorescent dye, which can have various industrial applications [38]. There are only a few reports on the production of this compound from actinobacteria, including two recent reports from *Streptomyces* [39, 40], but no information on the biosynthetic pathway of this compound is available. Other compounds included two compounds with the UV spectra both showing the absorption maxima at 222 nm and 278 nm and a molecular mass of 374.0 and 375.0 respectively, and another one with a UV spectrum showing the absorption maximum at 226 and 289 nm and a molecular mass of 519.1 (Additional file 5: Figure S4), all of which could not be matched with any known compounds and thus need further characterization. The results of metabolite analysis clearly showed the potential of strain CN732 as a producer of novel metabolites.

### Comparative genome analysis

#### Comparison of biosynthetic gene clusters with other Streptomyces

The biosynthetic potential of *S. yeochonensis* CN732 was compared with 14 non-acidophilic *Streptomyces* species for which high quality genome data are available and also with four other acidophilic *Streptomyces* species [41] (Table 1). Although a great diversity of BGCs were observed in all of the genomes, few secondary metabolite producing gene clusters such as melanin, Type 1 PKS-NRPS, siderophore, and Type 3 PKS of strain CN732 exhibited higher prevalence in all acidophilic genomes (Table 3). While the former two shared limited similarity (< 13%) with the known istamycin and meilingmycin BGCs, high levels of similarity (> 80%) were observed for the latter two clusters with desferrioxamine B and alkylresorcinol BGCs, respectively. In *S. avermitilis*, two melanin BGCs are present and each is composed of two main genes, *MelC1* (tyrosinase cofactor) and *MelC2* (tyrosinase) [42] in addition to few other genes [43]. Both genes were present in the melanin BGCs of all 5 acidophilic species including CN732 and 3 other S*treptomyces* species (that also showed similarity to istamycin BGC). One significant difference observed in these melanin BGCs was that a group 1 glycosyl transferase (GT1) was present in those of all acidophilic species, which is absent in *S. avermitilis* and other *Streptomyces* except *S. collinus* Tu 365.

**Table 3:** Over-representation of known biosynthetic gene clusters present in 5 neutrotolerant acidophilic *Streptomyces* and their comparison with 14 non-acidophilic *Streptomyces* species used in this study. Known BGC that were present in *S. yeochonensis* CN732 and at least in two or more neutrotolerant genomes were considered

Cluster type	Most similar known cluster	MIBiG BGC-ID	Distribution (%)	
			Acidophilic	Non-acidophilic
Siderophore	Desferrioxamine B	BGC0000941_c1	80	28.6
T2PKS	Spore pigment	BGC0000271_c1	100	71.4
Melanin	Istamycin	BGC0000700_c1	100	21.4
T1PKS-NRPS	Meilingmycin	BGC0000093_c1	80	28.6
Terpene	2-methylisoborneol	BGC0000658_c1	40	35.7
T3PKS	Alkylresorcinol	BGC0000282_c1	80	14.3
Terpene	Hopene	BGC0000663_c1	100	92.9

#### Core genes specific to acidophilic species

To check any significant differences between the core genomes of acidophilic and non-acidophilic *Stretomyces* species, the core genomes for these two datasets were identified by using BPGA [44] pipeline. A total of 1869 genes (which is similar to 1797 genes detected by EDGAR) represented the core genome of non-acidophilic species. Similarly, 2796 genes consisted of the core genome of 5 acidophilic species. Comparison of these two core genomes led to the identification of at least 89 genes that were specific to the core genome of non-acidophilic species (subset 1), whereas about 340 genes were exclusively present in acidophilic core genome only (subset 2). Despite the similarities in the enrichment of KEGG pathways in both core genomes, a significant difference in these two subsets was observed. Only 13 statistically significant (*p*-value < 0.05) KEGG pathways were enriched in subset 1, whereas subset 2 exhibited at least 26 pathways. Specifically, one of the highly enriched pathways observed in the subset 2 was that of “microbial metabolism in diverse environments (map 01120)”. Other pathways enriched in this subset include pathways related to the metabolism of amino acids (valine, leucine, isoleucine, and arginine), ABC transporters, base excision repair and DNA replication. In contrast, subset 1 was over-represented with pathways focusing on biosynthesis of secondary metabolites. Similarly, a compelling difference in the over-representation of various gene ontology (GO) terms was observed between two subsets. A high number of 106 statistically significant GO terms were detected for subset 2 as compared to only 36 for subset 1. Subset 1 was enriched with several processes related to response to stimulus (e.g. stress and DNA damage) and nuclease activities. Contrastingly, subset 2 exhibited over-representation of processes related to cell and carbohydrate metabolism. Studies have shown that the genes associated with processes related to lipids, carbohydrates and amino acid metabolism regulates the cell survival process under stress conditions [45].

#### *S*. *yeochonensis* CN732 specific CAZy genes

The genome of CN732 contained 1320 unique CDS when compared with those of other *Streptomyces* species. While majority (79%) of these singletons represented hypothetical proteins, several enzymes of significant importance were also observed. These include CAZymes such as alpha/beta hydrolases, beta-galactosidases, beta-glucosidases, and others with CAZy domains including glycosyl hydrolases (GHs) and glycosyltransferases (GTs).

At least 88 CAZy domains in 67 singletons were observed, suggesting the presence of one or more such domains in these singletons (Table 4). The most abundant domain found in 8 singletons represented GH3 family of β-glucosidases (BGs). In addition to their roles in cellulose modifications [46, 47], BGs have been of significant interest because of their industrial applications including flavor and aroma production, and the release of aromatic compounds from wine, fruit juices and flavorless products [48]. In addition to BGs, other families of cellulose degrading enzymes identified in these singletons include one copy each of GH8 and GH12 CAZymes. The hydrolysis of β-1,4-glycosidic bonds present in cellulose, chitosan, and xylan is catalyzed by the enzymes of GH8 family [49]. The enzymes of GH12 family are also multi-functional, with most of the members exhibiting endoglucanase activity. However, activities against xyloglucan, β-1,3-1,4-glucan and xylan are also observed [50]. Some members of GH12 family show extreme range of optimum temperatures [51, 52] and pH values [53]. These properties make the enzymes of this family strong candidates for industrial applications [54].

**Table 4:** List of *S. yecochonensis* CN732 specific (singletons) CAZy domains and their known activities or carbohydrate-binding capabilities

CAZy Family	Known Activities^a^	Count
Glycosyl hydrolases		
GH3	β-Glucosidase; xylan 1,4-β-xylosidase; β-glucosylceramidase	8
GH109	α-N-Acetylgalactosaminidase	5
GH78	α-L-Rhamnosidase; rhamnogalacturonan α-L-rhamnohydrolase; L-Rhap-α-1,3-D-Apif -specific α-1,3-L-rhamnosidase	5
GH18	Chitinase; lysozyme; endo-β-N-acetylglucosaminidase	3
GH95	α-L-Fucosidase; α-1,2-L-fucosidase; α-L-galactosidase	3
GH2	β-Galactosidase; β-mannosidase; β-glucuronidase	2
GH32	Invertase; endo-inulinase; β-2,6-fructan 6-levanbiohydrolase	2
GH42	β-Galactosidase; α-L-arabinopyranosidase	2
GH87	Mycodextranase; α-1,3-glucanase	2
GH10	Endo-1,4-β-xylanase; endo-1,3-β-xylanase; tomatinase	1
GH106	α-L-Rhamnosidase	1
GH12	Endoglucanase; xyloglucan hydrolase; β-1,3-1,4-glucanase	1
GH121	β-L-Arabinobiosidase	1
GH130	β-1,4-Mannosylglucose phosphorylase; β-1,4-mannooligosaccharide phosphorylase; β-1,4-mannosyl-N-acetyl-glucosamine phosphorylase	1
GH139	α-2-O-Methyl-L-fucosidase	1
GH141	α-L-Fucosidase; xylanase	1
GH27	α-Galactosidase; α-N-acetylgalactosaminidase; isomalto-dextranase	1
GH35	β-Galactosidase; exo-β-glucosaminidase; exo-β-1,4-galactanase	1
GH4	Maltose-6-phosphate glucosidase; α-glucosidase; α-galactosidase	1
GH8	Chitosanase; cellulase; licheninase	1
GH92	Mannosyl-oligosaccharide α-1,2-mannosidase; mannosyl-oligosaccharide α-1,3-mannosidase; mannosyl-oligosaccharide α-1,6-mannosidase	1
Glycosyltransferases		
GT2	Cellulose synthase; chitin synthase; dolichyl-phosphate β-D-mannosyltransferase	5
GT4	Sucrose synthase; sucrose-phosphate synthase; α-glucosyltransferase	5
GT1	UDP-glucuronosyltransferase; zeatin O-β-xylosyltransferase; 2-hydroxyacylsphingosine 1-β-galactosyltransferase	1
GT27	Polypeptide α-N-acetylgalactosaminyltransferase	1
GT28	1,2-Diacylglycerol 3-β-galactosyltransferase; 1,2-diacylglycerol 3-β-glucosyltransferase; UDP-GlcNAc: Und-PP-MurAc-pentapeptide β-N-acetylglucosaminyltransferase	1
Carbohydrate esterases		
CE2	Acetyl xylan esterase	1
CE3	Acetyl xylan esterase	1
CE14	N-Acetyl-1-D-myo-inosityl-2-amino-2-deoxy-α-D-glucopyranoside deacetylase; diacetylchitobiose deacetylase; mycothiol S-conjugate amidase	1
Carbohydrate-binding module (CBM)		
CBM13	Galactose, GalNAc, Mannose, Xylan	6
CBM32	Galactose, Lactose, LacNAc (β-D-galactosyl-1,4-β-D-N-acetylglucosamine), Polygalacturonic acid	4
CBM66	Fructans	3
CBM2	Cellulose, Chitin, Xylan	2
CBM35	Xylan (Ca dependent), Mannans and Mannooligosaccharides, β-galactan	2
CBM40	Sialic acid	2
CBM16	Cellulose, Glucomannan	1
CBM3	Cellulose, Chitin	1
CBM5	Chitin	1
CBM51	Galactose, Blood group A/B-antigens	1
CBM6	Cellulose, β-1,4-xylan, β-1,3-glucan, β-1,3-1,4-glucan, β-1,4-glucan	1
CBM63	Cellulose	1
CBM67	L-rhamnose	1
CBM9	Cellulose	1
Auxiliary activities (AA)		
AA3	Cellobiose dehydrogenase; glucose 1-oxidase; aryl alcohol oxidase	1

^a^ In case of multiple activities shown by a certain domain, only the first three reported in CAZy database are shown

Another class of biotechnologically important enzymes that were abundant and unique to CN732 were α-L-rhamnosidases. On the basis of the sequence similarity, α-L-rhamnosidases are grouped into three distinct GH families, viz., GH28, GH78, and GH106 [10]. Five singletons of family GH78 and one belonging to GH106 were identified in CN732 genome. Three out of the 5 GH78 enzymes are associated with one carbohydrate binding module (CBM) representing either CBM32, CBM35 or CBM66. The CBMs are known to improve the enzyme-substrate association [55]. Contrastingly, GH106 enzyme consisted of CBM67 domain. Overall, at least 14 different types of CBMs were observed in the 67 singletons. Until now, only a limited number of GH78 α-L-rhamnosidases have been experimentally characterized and most of them have been reported from lactic acid bacteria [56]. Six α-*N*-acetylgalactosaminidases including 5 GH109 and 1 GH27 family were also identified. The enzyme belonging to GH109 has been successfully used for the removal of A antigen on red blood cells, therefore, initiating the prospects of converting the blood groups to universal group O [57].

Three singletons were annotated as GH18 family chitinases, each of which contained one carbohydrate binding module. Among the different types of chitin degrading enzymes, family GH18 enzymes are considered as the central ones responsible for the bioconversion of crystalline chitin [58].

At least three singletons belonging to GH95 family of fucosidases were also identified. The discovery of new fucosidases with high regiospecificity and broad characteristics are thought to be of great significance towards analytical or biosynthetic applications [59].

Moreover, at least 13 glycosyltransferases (GT) belonging to five different families were also detected. GTs are the enzymes involved in the biosynthesis of disaccharides, oligosaccharides and polysaccharides by catalyzing the transfer of sugar moieties from activated donor molecules to specific acceptor molecules [60]. Three singletons were also annotated to possess carbohydrate esterase (CE) domains. CEs catalyze the de-O or de-N-acylation of substituted saccharides by removing their ester decorations, and thus have considerable significance as biocatalysts in a range of biotechnological applications [61].

The presence of several important CN732 specific CAZymes highlights the promising potential of this strain for breakdown of biopolymers. The original study by Seong [62] also indicated that strain CN732 was capable of utilizing or degrading a variety of substrates, including oligosaccharides or polysaccharides such as starch, melezitose, inulin and starch, and various others such as salicin, gluconic acid, 2-keto-D-gluconic acid, ribitol, sorbitol, nicotinamide, malic acid, malonic acid, oxalic acid and succinic acid.

### Sortases in the biosynthetic gene clusters of strain CN732

**Fig. 4:**
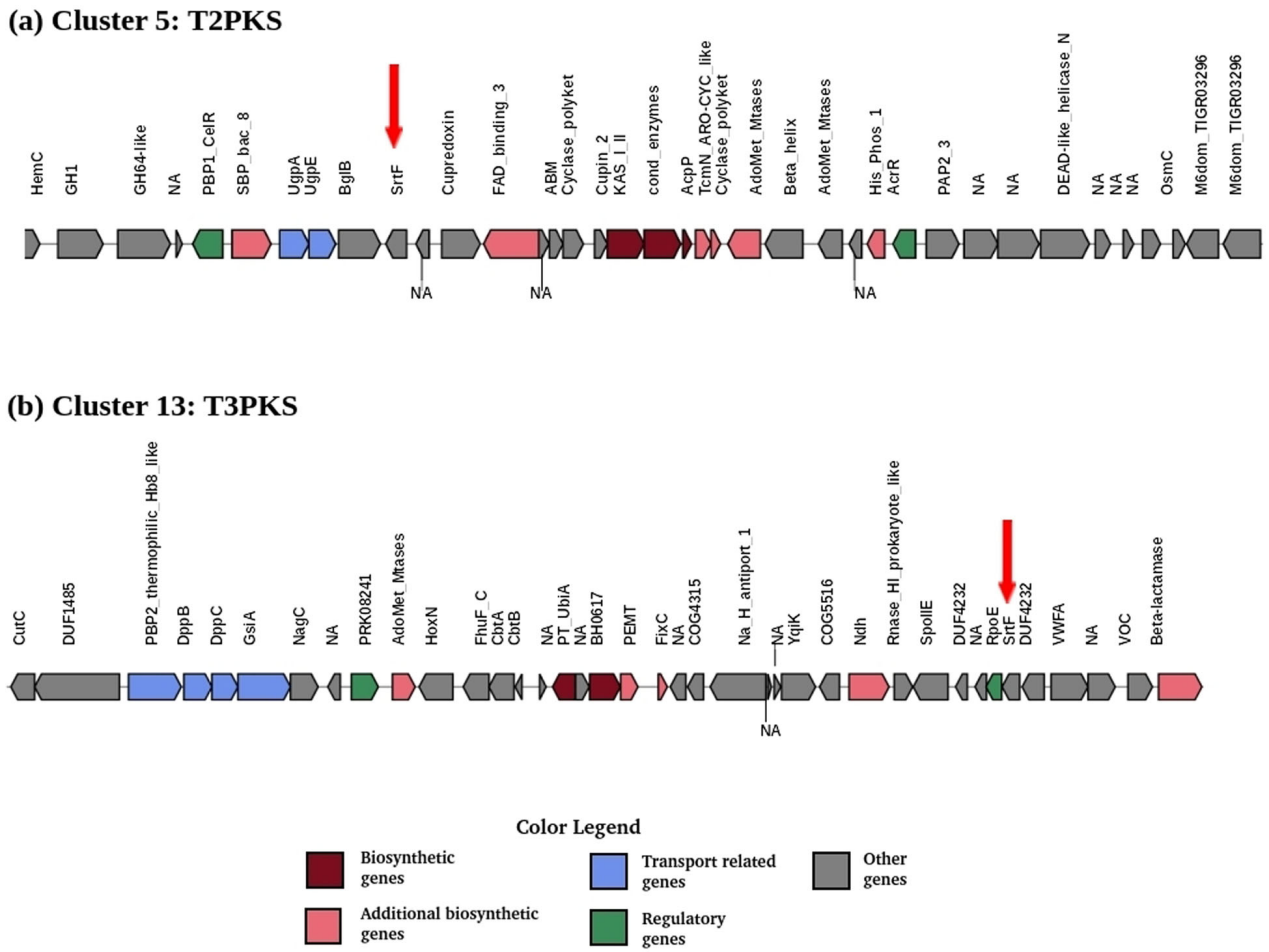
antiSMASH predicted biosynthetic gene clusters showing the presence of class F sortase enzymes (indicated by red arrows) in **a** Type 2 PKS cluster 5, and **b** Type 3 PKS cluster 13. While cluster 5 is enriched with CAZymes such as GH1, GH64, and Beta-glucosidase (BglB), several genes that are involved in metal resistance including *CutC* family (Copper transport), ABC-type dipeptide/oligopeptide/nickel transport system genes (*DppB*, *DppC*), and *HoxN* (high-affinity nickel permease) were observed in cluster 13

Out of 1320 singletons observed in CN732, one represented a class E sortase. Sortases are cysteine transpeptidases which covalently link proteins to their cell wall and play a crucial role in regulating the surface architecture of Gram-positive as well as few species of Gram-negative bacteria [63–65]. The highly conserved histidine (HIS), cysteine (CYS) and arginine (ARG) required for the catalytic activity of sortases [66, 67] were conserved in this singleton. In addition to this singleton and 3 other core genome sortases, two genes that encode class F sortase enzymes were also detected in the predicted PKS type BGCs, cluster 5 (Type 2 PKS) and 13 (Type 3 PKS) (Fig. 4). Among the conserved catalytic residues, HIS and CYS were present whereas ARG was replaced by asparagine (ASN) and reflect our recent analysis that in case of class F enzymes, ARG is replaced by ASN in Actinobacteria [68]. At present, six different classes (A-F) of sortase enzymes are recognized [64] with no function designated to class F enzymes. The current study is the first which reports the identification of class F enzymes in BGCs for secondary metabolites, and hence indicating their potential roles. Only 4 class E and 3 (out of which 2 were detected in antiSMASH clusters, Fig. 4) class F sortases were present in CN732 genome (Table 5). However, when compared with the distribution of sortase enzymes in other strains, the number of sortases varied and occurred within a range of 6–11 with an average of about 8 sortases per genome. These results are consistent with our recent work on sortase superfamily [68]. The genomes of other *Streptomyces* strains were scanned to further explore the presence of putative sortase substrates that might help in deciphering the functional roles of sortases, and a total of 126 (including 5 from CN732) putative substrates for sortases that carry the Gram-positive anchor domain (pf00746) were identified from all genomes. To further check if sortase substrates are also present in the biosynthetic gene clusters, BLAST search of these 126 potential sortase substrates was carried out against the amino acid sequences available at MIBiG (Minimum Information about a Biosynthetic Gene cluster) [69] database. Interestingly, five substrate sequences belonging to the DUF320 superfamily were identified. These sequences exhibited about 65% similarity to a putative small membrane protein present in the lactonamycin BGC from *Streptomyces rishiriensis* [70]. Three out of these 5 sequences consisted of LAETG motif whereas the remaining 2 consisted of LAHTG recognition motifs, suggesting that they are class E substrates. Taken together the identification of sortase enzymes and their substrates in BGCs suggest their potential involvement in secondary metabolism.

**Table 5:** Distribution of sortases among *Streptomyces* genomes used in this study

PATRIC Genome ID	BioProject Accession no.	Genome Name	Number of		Total
			Sortase E	Sortase F	
1888.4	PRJNA271625	*S. albus* DSM 41398	4	2	6
227882.9	PRJNA189	*S. avermitilis* MA-4680	5	4	9
749414.3	PRJNA46847	*S. bingchenggensis* BCW-1	3	4	7
100226.15	PRJNA242	*S. coelicolor* A3(2)	5	2	7
1214242.5	PRJNA171216	*S. collinus* Tu 365	5	4	9
1214101.3	PRJEB184	*S. davawensis* JCM 4913	6	4	10
1303692.3	PRJNA192408	*S. fulvissimus* DSM 40593	6	4	10
1907.4	PRJNA260814	*S. glaucescens* GLA.O	4	2	6
455632.4	PRJNA20085	*S. griseus* NBRC 13350	5	4	9
40318.3	PRJNA259817	*S. nodosus* ATCC 14899	5	3	8
1343740.8	PRJNA207502	*S. rapamycinicus* NRRL 5491	3	4	7
862751.12	PRJNA38225	*Streptomyces* sp. SirexAA-E	6	3	9
953739.5	PRJNA62209	*S. venezuelae* ATCC 10712	7	4	11
362257.4	PRJNA244969	*S. vietnamensis* GIM4.0001	7	4	11
1449355.3	PRJNA234789	*S. yeochonensis* CN732	4	3	7

## Conclusion

In this study, the genome of *Streptomyces yeochonensis* CN732 was sequenced and thoroughly annotated. Prediction of biosynthetic gene clusters for secondary metabolites suggested that this acidophilic actinobacterial strain has the potential to produce novel metabolites, which could be of industrial or scientific significance but have not been identified properly yet. Specifically, four biosynthetic clusters including two hybrid Type 1 PKS-NRPS, one Type 1 PKS-butyrolactone and one NRPS clusters were predicted with a core structure, each representing a putative secondary metabolite. One notable feature was that the majority of BGCs shared similarity, albeit at low levels, with those producing anticancer compounds, which were then followed by antibacterial and antiparasitic compounds. Comparative genomic analysis with other *Streptomyces* revealed several genes specific to strain CN732, and also those specific to acidophilic species of *Streptomyces*, which may help them thrive in harsh environmental conditions. These singletons included biosynthetic genes such as NRPS and PKS, various carbohydrate active enzymes and cysteine transpeptidases (sortases). In addition to their potential to degrade biomass, the CAZymes identified in various biosynthetic clusters may also represent interesting candidates to manipulate the structures of various biosynthetic compounds. One of the interesting outcomes of this study was the discovery of at least two class F sortases present in biosynthetic gene clusters, suggesting their direct or indirect role in secondary metabolite production. At present, the exact function of class F sortases is unknown, and therefore it will be interesting to identify and explore the potential role of these enzymes in well-known biosynthetic clusters. Current efforts are being made to investigate the biosynthetic as well as the biomass-degrading capabilities of this *Streptomyces* strain.

## Methods

### Strain and cultivation

Strain CN732 is the type strain of *Streptomyces yeochonensis* [18]. The strain was cultivated on acidified ISP (International *Streptomyces* Project) medium 2 (glucose 0.4%, yeast extract 0.4%, malt extract 1%, pH adjusted to 5.0) agar or broth at 30 °C.

### Genome sequencing and assembly

For the genome scale study as well as comparative genomic analysis to examine the biosynthetic and enzymatic potential of *S. yeochonensis* CN732, the whole genome of the strain was analyzed to a permanent draft level. The biomass of CN732 for genome analysis was obtained from the culture grown at 30 °C for 3 days in ISP (International *Streptomyces* Project) 2 (glucose 0.4%, yeast extract 0.4%, malt extract 1%) broth. A high quality genomic DNA was prepared using DNA prep kit (Solgent, Republic of Korea), and DNA quantification was by PicoGreen dsDNA reagent kit (Thermo, USA). The genomic DNA was sequenced at DOI Joint Genome Institute (JGI) using PacBio [71] RS, PacBio RS II sequencing method (http://allseq.com/knowledge-bank/sequencing-platforms/pacific-biosciences) and is available under the Project ID: 1030660. The genome data was also submitted to NCBI under the accession number PRJNA234789. HGAP v. 2.2.0.p1 [72] was used to assemble the raw reads.

### Genome annotation and bioinformatics analysis

Rapid Annotation using Subsystem Technology v.2.0 server (RAST) [73] was used for genome annotation. The coding sequences were further annotated using the standalone version of HMMER v3.1b2 (http://hmmer.org/) and by downloading all HMM models for bacteria from eggNOG v4.5.0 [74]. Additionally, the predicted proteins were assigned to orthologous groups and mapped to KEGG pathways by using the KEGG Automatic Annotation Server (KAAS) [75]. Statistical enrichment of core genes in KEGG pathways and gene ontology (GO) processes was carried out using KOBAS software [76]. antiSMASH [77] was used to predict the gene clusters that may have potential for the production of secondary metabolites.

### PCR amplification for NRPS and PKS I genes

The NRPS and PKS I genes were amplified by PCR using following primers, A3F (5′-GCSTACSYSATSTACACSTCSGG-3′)/A7R (5′-SASGTCVCCSGTSCGGTAS-3′) and K1F (5′-TSAAGTCSAACATCGGBCA-3′)/M6R (5′-CGCAGGTTSCSGTACCAGTA-3′) [29]. Reactions for NRPS genes were performed in a final volume of 20 μl containing 2 μl of extracted DNA, 1 μl of each primer (10 pmol), 0.4 μl of 10 mM dNTPs mixture (Solgent), 2 μl of 10X buffer for polymerase, and 0.1 μl of *Taq* polymerase (Solgent) with 13.5 μl of distilled water. Reactions for PKS 1 genes were performed in a final volume of 20 μl containing 2 μl of extracted DNA, 0.8 μl of each primer (10 pmol), 0.4 μl of 10 mM dNTPs mixture (Solgent), 2 μl of 10X buffer for polymerase, 0.08 μl of *Taq* polymerase (Solgent), and 2 μl of DMSO with 11.92 μl of distilled water. Amplification processes were then performed in a BIOER Gene Pro Thermal Cycler TC-E-48D, according to the following conditions: 5 min at 95 °C and 35 cycles of 30 s at 95 °C, 2 min at 55 °C, 59 °C for A3F/A7R or 58 °C for K1F/M6R, and 4 min at 72 °C, followed by 10 min at 72 °C [29]. Amplicons were analyzed by electrophoresis in 2% (w/v) agarose gels stained with ethidium bromide, and also purified by using a PCR purification kit (Macherey-Nagel).

### Test of antimicrobial activity

The antimicrobial potential of CN732 was carried out against 4 Gram positive bacteria, 7 Gram negative bacteria and 2 yeast species using 5 different media (Additional file 2: Table S2). The suspensions of CN732 were spotted on each agar plate and incubated at 30 °C for 1 week, then the test microbes mixed with soft agar (0.8%) were overlayed on the plate and the plate incubated for 1~3 days to examine the formation of clear zones.

### HPLC-MS analysis of metabolites from *S. yeochonensis* CN732

*S. yeochonensis* CN732 was initially cultivated in 50 mL of acidified ISP medium 2 (pH 5). After the strain was cultivated for 4 days on a rotary shaker at 170 rpm and 30 °C, 10 mL of the culture was transferred in 1 L of modified Bennet’s medium in a 2.8-L Fernbach flask. The entire culture (72 L) was extracted twice with ethyl acetate (150 L). The EtOAc extract was concentrated in vacuo to yield 10 g of dry material.

Optical rotations were measured using a JASCO P-1020 polarimeter. UV spectra were acquired on a Chirascan plus spectrometer of Applied Photophysics Ltd. Low-resolution electrospray ionization source mass spectra were acquired with an Agilent Technologies 6130 quadrupole mass spectrometer coupled to an Agilent Technologies 1200 series high-performance liquid chromatography (HPLC) instrument.

### Comparative genomic analysis

The complete 16S rRNA sequences for all genomes used in this study were predicted from their genomic data by using local installation of RNAmmer [78]. Although there are several genomes available for the *Streptomyces* in the databases and analyzing these many genomes requires rich computational resources. Therefore, 14 genomes of neutrophilic species, well known either for their secondary metabolite biosynthesis such as *S. coelicolor* [79] and *S. avermitilis* [80], or biomass-degrading properties including *Streptomyces* sp. SirexAA-E [8] (Table 1), were selected along with 4 genomes of neutrotolerant acidophilic species [41]. Their 16S rRNA sequences were aligned with ClustalW tool in the MEGA version 7 software [81], and a neighbor-joining tree was constructed by using a bootstrap test of 1000 replicates. Moreover, a whole genome based phylogeny was inferred using the TYGS webserver [82]. The average nucleotide identity (ANI) values across all 19 *Streptomyces* genomes were calculated by using orthoANIu [83]. Comparative genomic analysis to identify CN732 specific genes (singletons), as well as core-genome was carried out by requesting an access to the private project in EDGAR (Efficient Database framework for comparative Genome Analyses using BLAST score Ratios) software [84].

To annotate sortase sequences in all *Streptomyces* genomes, position-specific scoring matrix (PSSM) searches against pre-formatted CDD [85], “little_endian” (Downloaded: 12th July 2017) were carried out by using a standalone RPS-BLAST (v2.6.0+) algorithm. Putative sortase substrates were identified in each genome by scanning all the protein coding sequences (CDS) with the HMMSEARCH function within HMMER package using hidden Markov models from Pfam [86] database for the family “Gram-positive anchor” (pf00746). dbCAN [87] server was used to annotate the CN732 specific CAZymes. Hits with an e-value threshold of ≤10^− 5^ were considered only.

## Supplementary information

**Additional file 1: Figure S1.** Phylogenetic tree of *S. yeochonensis* CN732 and other *Streptomyces* based on predicted 16S rRNA sequences extracted from respective genomes. The bootstrap consensus tree was inferred from 1000 replicates using the neighbor-joining method. The evolutionary distances were computed using the Jukes-Cantor method. *Kitasatospora setae* KM-6054 and *Streptacidiphilus albus* JK-83 were added as outgroups.

**Additional file 2: Table S1.** antiSMASH clusters with CAZy domains and their known activities. **Table S2.** Antimicrobial test results of *S. yeochonensis* CN732 in various culture media.

**Additional file 3: Figure S2.** PCR-based detection of NRPS and PKS 1 genes for strain CN732. (a), NRPS; (b), PKS 1.

**Additional file 4: Figure S3.** Characteristics of lumichrome, a representative metabolite from strain CN732 based on HPLC-MS analysis. (a), UV-visible spectrum; (b), positive ion mass spectrum; (c), structural formula.

**Additional file 5: Figure S4.** Characteristics of unidentified metabolites (a-c) from strain CN732 based on HPLC-MS analysis.

## Data Availability

The whole genome data are available at DDBJ/ENA/GenBank under the bioproject accession PRJNA234789 (https://www.ncbi.nlm.nih.gov/bioproject/234789).

## Abbreviations

CAZyme: carbohydrate active enzyme
NRPS: non-ribosomal peptide synthetase
PKS: polyketide synthase
