# Supplementary figures and images for: Genome-based analysis for the bioactive potential of Streptomyces yeochonensis CN732, an acidophilic filamentous soil actinobacterium
Source: BMC Genomics. 2020 Feb 3;21:118. doi: 10.1186/s12864-020-6468-5 (PMC6998099; doi:10.1186/s12864-020-6468-5)

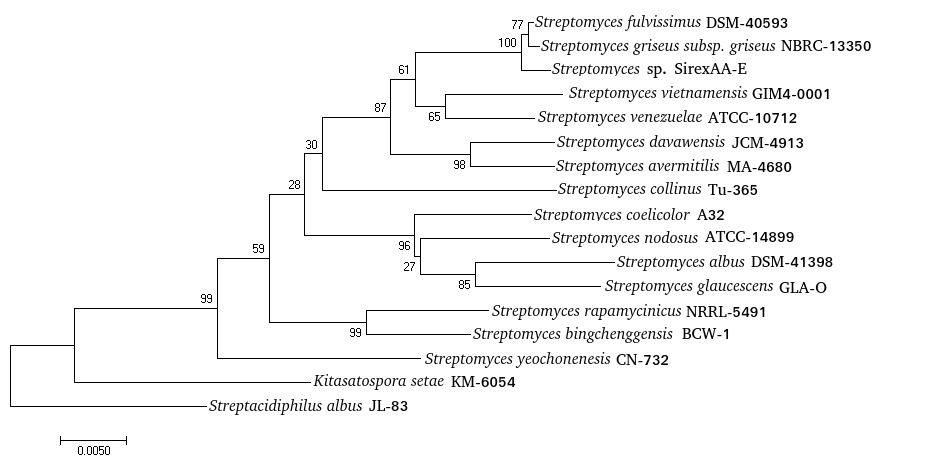

Supplement: Supplementary file 1 — Additional file 1: Figure S1. Phylogenetic tree of S. yeochonensis CN732 and other Streptomyces based on predicted 16S rRNA sequences extracted from respective genomes. The bootstrap consensus tree was inferred from 1000 replicates using the neighbor-joining method. The evolutionary distances were computed using the Jukes-Cantor method. Kitasatospora setae KM-6054 and Streptacidiphilus albus JK-83 were added as outgroups. [file 12864_2020_6468_MOESM1_ESM.tif]

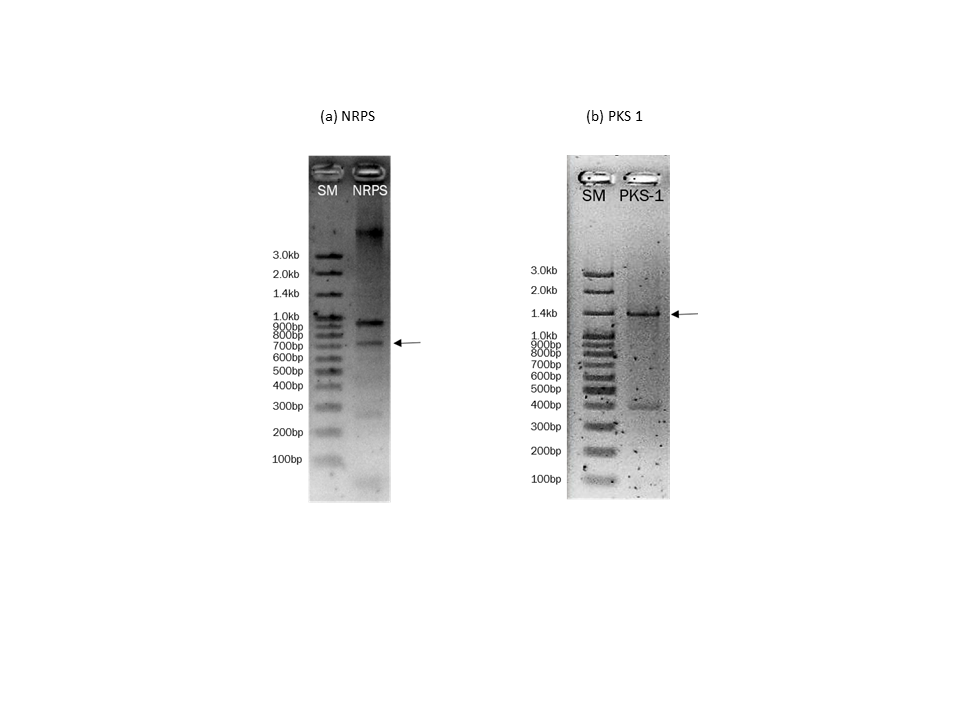

Supplement: Supplementary file 3 — Additional file 3: Figure S2. PCR-based detection of NRPS and PKS 1 genes for strain CN732. (a), NRPS; (b), PKS 1. [file 12864_2020_6468_MOESM3_ESM.tif]

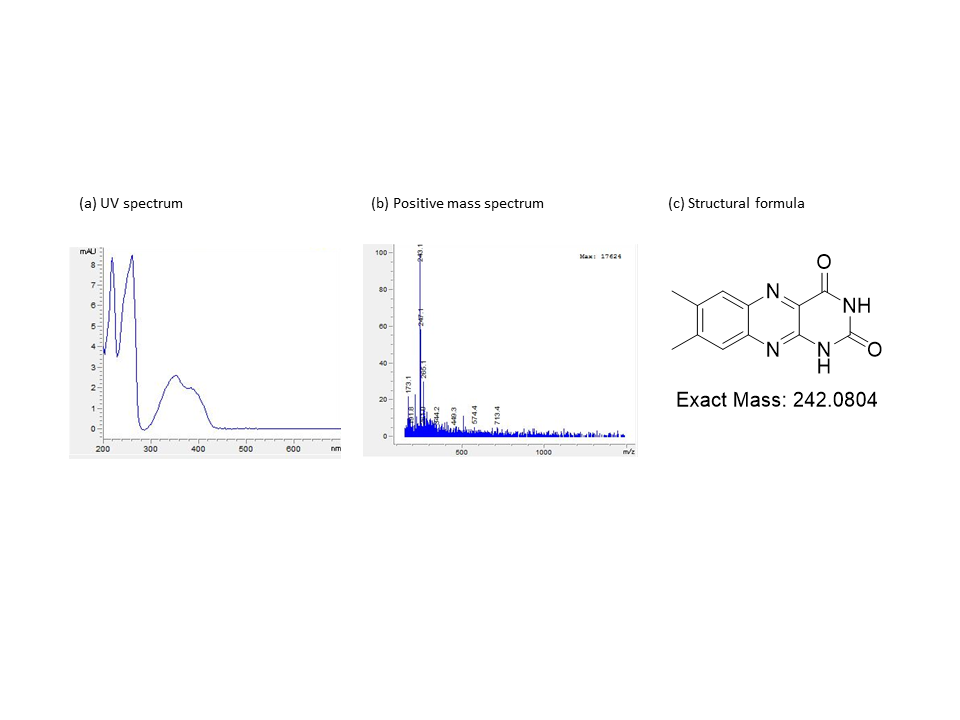

Supplement: Supplementary file 4 — Additional file 4: Figure S3. Characteristics of lumichrome, a representative metabolite from strain CN732 based on HPLC-MS analysis. (a), UV-visible spectrum; (b), positive ion mass spectrum; (c), structural formula. [file 12864_2020_6468_MOESM4_ESM.tif]

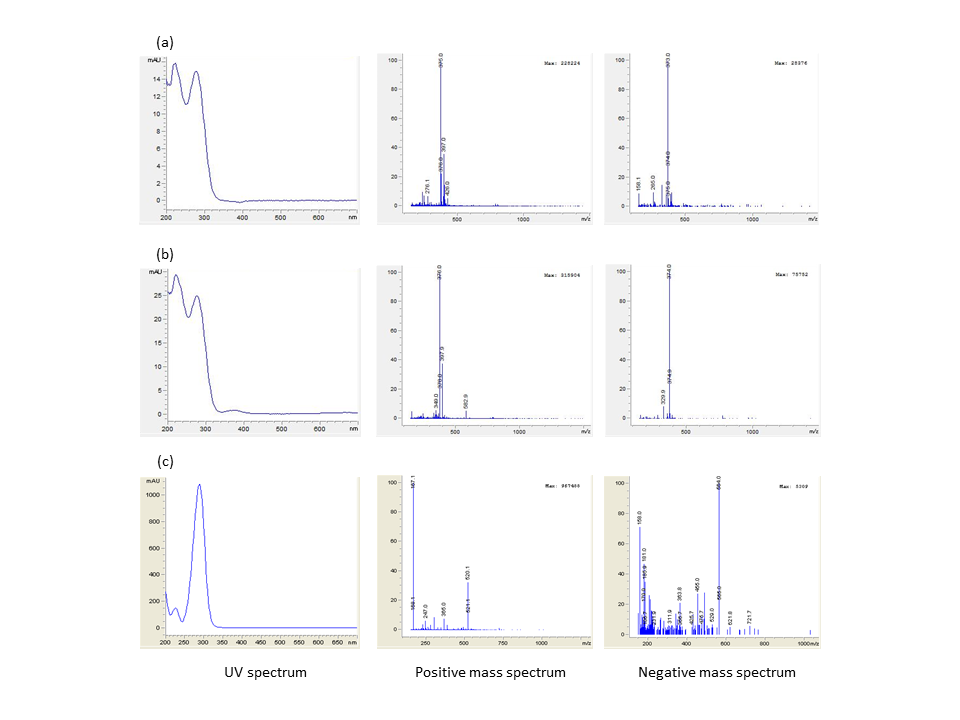

Supplement: Supplementary file 5 — Additional file 5: Figure S4. Characteristics of unidentified metabolites (a-c) from strain CN732 based on HPLC-MS analysis. [file 12864_2020_6468_MOESM5_ESM.tif]
