# Supplementary material for: Genome-based analysis for the bioactive potential of Streptomyces yeochonensis CN732, an acidophilic filamentous soil actinobacterium
Source: BMC Genomics. 2020 Feb 3;21:118. doi: 10.1186/s12864-020-6468-5 (PMC6998099; doi:10.1186/s12864-020-6468-5)
Supplement: Supplementary file 2 — Additional file 2: Table S1. antiSMASH clusters with CAZy domains and their known activities. Table S2. Antimicrobial test results of S. yeochonensis CN732 in various culture media. [file 12864_2020_6468_MOESM2_ESM.docx]

**Table S1. AntiSMASH clusters with CAZy domains and their known activities**

In case there were more than one known activities for a CAZy family, only the first reported activity from CAZy database is shown. Clusters for which a structure was predicted are highlighted in bold.

| **Type** | **Cluster No.** | **CAZy Family** | **Known Activities** |
| --- | --- | --- | --- |
| Terpene | 10 | GT2 | Cellulose synthase |
|  | 14 | GT2  CE3 | Cellulose synthase  Acetyl xylan esterase |
|  | 20 | GH35; CBM51 | α-L-Rhamnosidase; galactose binding |
| **NRPS** | **2** | **GH2; CBM35** | **β-Galactosidase; xylan binding** |
|  |  | **GT51** | **Murein polymerase** |
|  | 18 | GH106; CBM67 | α-L-Rhamnosidase; L-rhamnose binding |
| Type 1 PKS | 19 | AA3 | Cellobiose dehydrogenase |
| Type 2 PKS | 5 | GH5; CBM2 | Endo-β-1,4-glucanase / cellulase; cellulose binding |
|  |  | CBM32 | Galactose and lactose binding |
|  |  | GH1 | β-Glucosidase |
| Butyrolactone | 12 | CE7 | Acetyl xylan esterase |
| **T1PKS-NRPS** | **7** | **GH3** | **β-Glucosidase** |
|  |  | **GH109** | **α-N-Acetylgalactosaminidase** |
| **T1PKS-NRPS** | **22** | **GT28** | **1,2-Diacylglycerol 3-β-galactosyltransferase** |
|  |  | **CE4** | **Acetyl xylan esterase** |
|  |  | **GH3; CBM6; CBM32** | **β-Glucosidase; cellulose binding; galactose and lactose binding** |
|  |  | **GT87** | **Polyprenol-P-Man: α-1,2-mannosyltransferase** |
| **T1PKS-Butyrolactone** | **15** | **AA7** | **Glucooligosaccharide oxidase** |
|  |  | **AA3** | **Cellobiose dehydrogenase** |
| Nucleoside | 3 | GH99 | Glycoprotein endo-α-1,2-mannosidase |
| Melanin | 6 | GH26 | β-Mannanase |
| Bacteriocin | 21 | GH16 | Xyloglucan:xyloglucosyltransferase |

**Table S2. Antimicrobial activity of *S. yeochonensis* CN732 on various culture media**

| Test microbe | Culture Medium* | | | | | |
| --- | --- | --- | --- | --- | --- | --- |
|  | TSA | SNA | ISP 2 | Bennett's | MH | PEC |
| **Gram positive bacteria** |  |  |  |  |  |  |
| *Micrococcus luteus* | - | - | - | - | - | + |
| *Staphylococcus aureus* | - | - | - | - | - | + |
| *Corynebacterium dightheriae* | - | + | + | - | - | - |
| *Bacillus subtilis* | - | + | - | - | - | - |
| **Gram negative bacteria** |  |  |  |  |  |  |
| *Klebsiella pneumoniae* | - | + | - | - | - | + |
| *Salmonella enterica* | - | + | - | - | - | + |
| *Serratia marcescens* | - | + | - | - | - | + |
| *Escherichia coli* | - | + | - | - | - | - |
| *Enterococcus faecalis* | - | - | + | - | - | - |
| *Enterovacter cloacae* | - | + | - | - | - | + |
| *Pseudomonas aeruginosa* | - | + | - | - | - | + |
| **Yeasts** |  |  |  |  |  |  |
| *Candida krusei* | - | - | + | - | - | - |
| *Candida albicans* | - | + | - | - | - | - |

* TSA, tryptic soy agar; SNA, synthetic nutrient poor agar; ISP 2, International *Streptomyces* medium 2; MH, Mueller-Hinton medium; PEC, pectin-malt extract-casamino acid medium.
